# Delaying Gal4-Driven Gene Expression in the Zebrafish with Morpholinos and Gal80

**DOI:** 10.1371/journal.pone.0016587

**Published:** 2011-01-26

**Authors:** Adèle Faucherre, Hernán López-Schier

**Affiliations:** Laboratory of Sensory Cell Biology and Organogenesis, Centre de Regulació Genòmica, Barcelona, Spain; Virginia Industries for the Blind, Belgium

## Abstract

The modular Gal4/UAS gene expression system has become an indispensable tool in modern biology. Several large-scale gene- and enhancer-trap screens in the zebrafish have generated hundreds of transgenic lines expressing Gal4 in unique patterns. However, the early embryonic expression of the Gal4 severely limits their use for studies on regeneration or behavior because UAS-driven effectors could disrupt normal organogenesis. To overcome this limitation, we explored the use of the Gal4 repressor Gal80 in transient assays and with stable transgenes to temporally control Gal4 activity. We also validated a strategy to delay Gal4-driven gene expression using a morpholino targeted to Gal4. The first approach is limited to transgenes expressing the native Gal4. The morphant approach can also be applied to transgenic lines expressing the Gal4-VP16 fusion protein. It promises to become a standard approach to delay Gal4-driven transgene expression and enhance the genetic toolkit for the zebrafish.

## Introduction

The Gal4 protein from yeast is a transcription factor that activates the expression of genes required for growth on galactose. It does so by binding to an Upstream Activating Sequence (UAS) located in the promoters of target genes. In the absence of galactose, Gal4 is inactive due to the activity of the repressor protein Gal80, which binds to a region of about 30 amino acids in the activation domain of Gal4, preventing its interaction with the transcriptional machinery [1], [2]. The Gal4/UAS system has been exploited to drive gene expression in animals other than yeast and was initially employed in *Drosophila* [3], [4]. Nowadays, nearly all research using a genetic approach in the fly are based on the Gal4/UAS gene-expression system [5]. Several strategies have been adopted to refine Gal4 activity in *Drosophila*. For example, Gal80 has been used to antagonize Gal4 activity in developing and adult flies [6], [7]. Also, temperature-sensitive mutant forms of Gal80 and Gal4 have been exploited along with ligand-based technologies to further enhance conditional control [7]. Although the native Gal4 is highly efficient in *Drosophila*, it is a weak activator of transgene expression in the zebrafish [8]. Thus, its transactivation efficiency has been enhanced by replacing the C-terminal activation domain of the native Gal4 with the highly acidic activation domain of the herpes simplex virus VP16 [9], [10]. The resulting Gal4-VP16 fusion protein retains the Gal4's DNA binding specificity for the UAS but is a vastly more efficient transcription factor. The improvement of the efficiency of transgenesis in the zebrafish has created the opportunity to generate a variety of transgenic lines bearing Gal4 activators or UAS-driven effectors [11], [12], [13]. Some of these transgenic lines have already been useful to analyze early development, but comparatively they have been less effective as tools to characterize biological processes during postembryonic or larval stages, including regeneration and behavior because the early expression of Gal4-driven effector genes may disrupt the normal development of the target tissues [13], [14], [15], [16], [17]. In the zebrafish, spatiotemporal control of the Gal4 activity can be achieved by a chemically inducible system based on the insect-specific ecdysone receptor (EcR) [18]. The chimaeric transactivator composed of the Gal4 fused to the EcR (GV-EcR) is able to regulate transcription only after the addition of the small molecule tebufenozide, thereby allowing a temporal control of the target gene expression. In addition, clonal expression of Gal4 can be achieved through the use of the recently developed MAZe technology. Clones can be induced through heat-shock promoter-driven CRE recombinase expression, which allows termporal control of Gal4 activity. However, this technique is not efficient to express the Gal4 in entire tissues or organs [19]. None of the hundreds of transgenic Gal4 driver lines currently available use the inducible form of the Gal4 or can be adapted to MAZe. To overcome this limitation, here we report two general methods to temporally control Gal4 activity in the zebrafish. We show that native Gal4 activity can be repressed using Gal80 in transient assays by mRNA injection and with a stable transgene expressing the Gal80 by heat shock. Gal4-VP16, however, could not be repressed with Gal80 because this fusion protein lacks the binding site for Gal80. Therefore, we used a morpholino targeted to Gal4-VP16 to delay its activity until larval stages.

## Results and Discussion

### Gal80 inhibits Gal4 activity in the zebrafish

**Figure 1:**
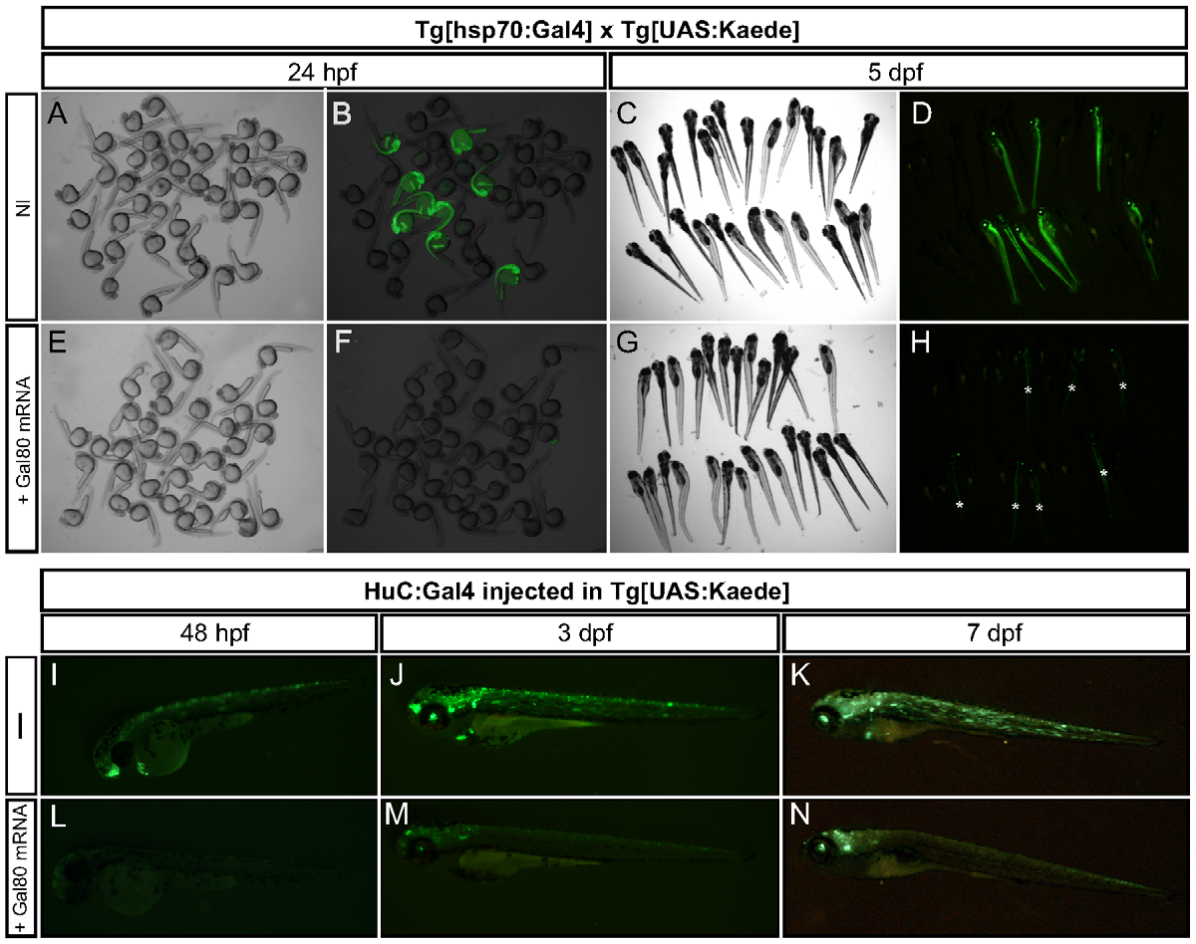
Gal80 expression inhibits Gal4 activity. (**A–H**) Embryos resulting from a cross between *Tg[hsp70:Gal4]* and *Tg[UAS:Kaede]* fish were either non-injected (NI, A–D) or injected with 100 pg of mRNA encoding full-length Gal80 (E–H). Representative specimens are depicted at 24 hpf (A–B, N = 18 GFP**^+^**/72 fish; E–F, N = 0 GFP**^+^**/75 fish) and 5 dpf (C–D, N = 18 GFP**^+^**/77 fish; G–H, N = 19 GFP**^+^**/82 fish). In H, asterisks indicate the fish displaying GFP. (**I–N**) Embryos resulting from a cross of *Tg[UAS:Kaede]* fish were injected with a DNA encoding full-length Gal4 under the control of the HuC promoter either alone (NI, I–K, N = 32) or with 100 pg of mRNA encoding full-length Gal80 (L–N, N = 37). Representative specimens are depicted at 48 hpf, 3 and 7 dpf.

To temporally control the activity of the Gal4, we used its natural repressor Gal80. We first tested whether ectopic Gal80 expression would interfere with the normal development of the zebrafish by injecting synthetic mRNA encoding the full-length Gal80 into fertilized eggs (Fig. 1). We used doubly-transgenic eggs derived from a cross between the *Tg[hsp70:Gal4]* transgenic line expressing the native form of Gal4 under the control of a heat-shock promoter and the *Tg[UAS:Kaede]* that expresses the Kaede photoconvertible protein upon Gal4 activity. At 10 hours post-fertilization (hpf), experimental and control embryos were subjected to a 30-minute heat-shock at 39°C to activate the expression of the Gal4. The following day, the resulting embryos were analyzed for gross anatomical defects and for the expression of green-fluorescent Kaede (Fig. 1A–H). In the non-injected control fish, we observed that a quarter of the population strongly expressed Kaede (Fig. 1A–B), which represents the expected ratio of offspring from heterozygous carrier parents. The embryos that developed from the Gal80 mRNA injected eggs grew normally, without any apparent defect and none expressed the fluorescent protein (Fig. 1E–F). However, at 5 days post-fertilization (dpf), the larvae derived from the Gal80 RNA injected eggs started to fluoresce, albeit with lower intensity than the non-injected controls (Fig. 1C–D, G–H). This result suggests that ubiquitous production of the Gal80 protein does not cause deleterious effects on zebrafish development and that it can efficiently inhibit the activity of the Gal4 in transgenic zebrafish. To ask whether Gal80's effect extends to specific cell types, we co-injected the mRNA encoding Gal80 and a DNA construct encoding the full-length Gal4 under the control of the HuC neural promoter in eggs from the stable *Tg[UAS:Kaede]* transgenic line [20] (Fig. 1I–N). We then assessed the resulting fish for green fluorescence and compared them with those injected with the HuC:Gal4 construct alone. At 48 hpf, the Gal80 expressing animals did not show any fluorescence, whereas control fish expressed Kaede in scattered neurons (Fig. 1I, L). The Gal80 RNA-injected embryos begun to express Kaede at 3 dpf, whose fluorescence peaked at 7 dpf, albeit less intensely than in control animals (Fig. 1J–K, M–N). These observations demonstrate that Gal80 is an efficient repressor of Gal4 in neural tissues. They also suggest that the gradual release of Gal4 inhibition likely due to the degradation of the Gal80 allows the expression of the UAS-driven genes at later stages of development with an average onset at 4 dpf.

### Gal4 inhibition by Gal80 is dose-dependent

**Figure 2:**
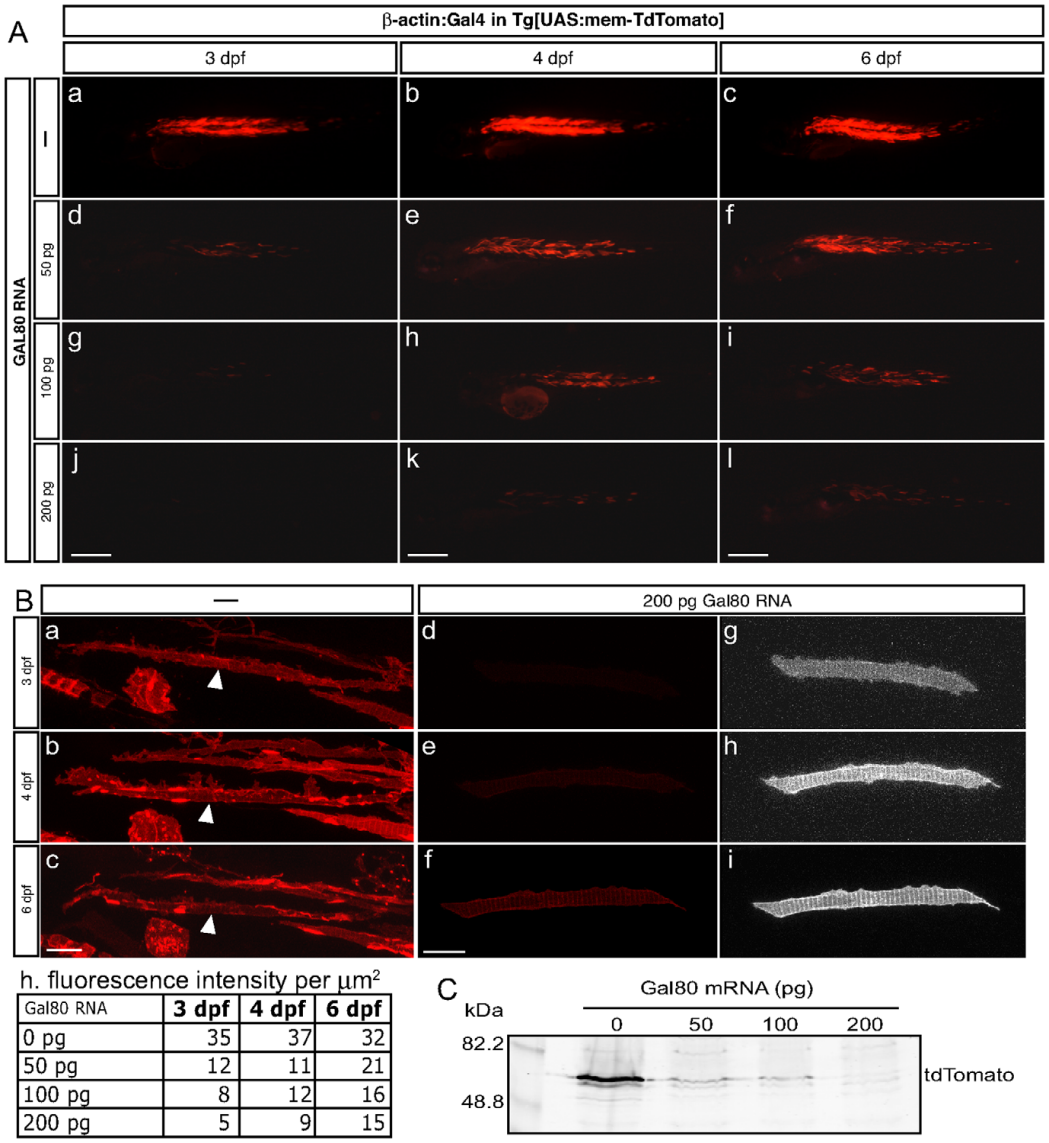
Gal80 RNA temporarily inhibits Gal4 activity in a dose-dependant manner. (**A**) Embryos resulting from a cross of *Tg[UAS:mem-TdTomato]* fish were injected with a DNA encoding full-length Gal4 under the control of the β-actin promoter alone (a–c) or with 50 pg (d–f, N = 21), 100 pg (g–i, N = 23) or 200 pg (j–l, N = 20) of mRNA encoding full-length Gal80. Representative specimens are depicted at 3, 4 and 6 dpf. (Scale bars: 300 µm). (**B**) Red fluorescence quantification of tdTomato expressing cells. (a–c) Maximal projection of muscle fibers from a embryo resulting from a cross of *Tg[UAS:mem-TdTomato]* fish injected with the β-actin:Gal4 construct alone. The white arrowheads indicate the quantified cell. (d–f) Maximal projection of muscle fibers from a embryo resulting from a cross of *Tg[UAS:mem-TdTomato]* fish injected with the β-actin:Gal4 construct and 200 pg of mRNA encoding full-length Gal80. Z-stacks have been captured at the same settings as in a–c. (g–i) Same as d-f with over-exposure. (Scale bars: 20 µm) (h) Quantitative values of fluorescence per µm^2^. Each value corresponds to the average of the mean values obtained for three different regions of each cell. (**C**) Anti-tdTomato western blotting of 3dpf fish resulting from a cross of *Tg[UAS:mem-TdTomato]* fish injected with the β-actin:Gal4 construct alone or with 50, 100 or 200 pg of mRNA encoding full-length Gal80.

**Figure 3:**
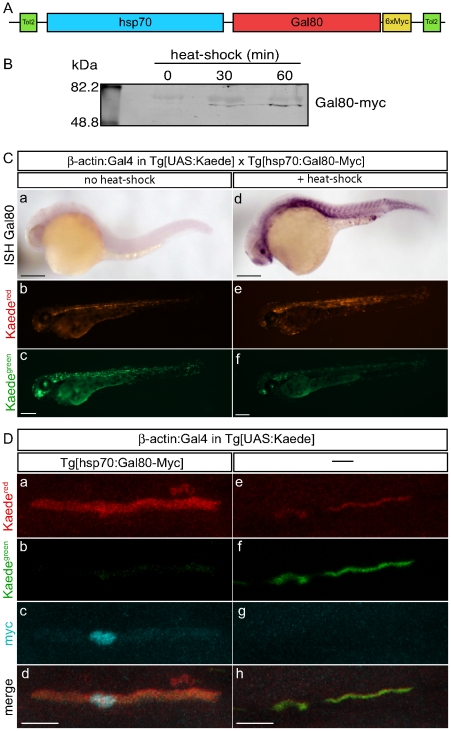
Temporal control of Gal80 expression. (**A**) Schematic representation of the construct hsp70:Gal80-myc. (**B**) Anti-myc western blotting of 48 hpf fish resulting from a cross of *Tg[hsp70:Gal80-myc]* fish after 0, 30 and 60 minutes of heat-shock. (**C**) Embryos resulting from a cross between *Tg[hsp70:Gal80-Myc]* and *Tg[UAS:Kaede]* fish were not submitted (a–c) or submitted to a one-hour heat-shock (d–f). Representative specimens are depicted for anti-Gal80 *in situ* hybridization at 30 hpf (a, d) or for Kaede expression at 2 dpf (b–c, d–e). (Scale bars: 150 µm). (**D**) Embryos resulting from a cross between *Tg[hsp70:Gal80-Myc]* and *Tg[UAS:Kaede]* fish were injected with a DNA encoding full-length Gal4 under the control of the β-actin promoter. At 24 hpf, the embryos were subjected to a 30-minute heat-shock at 39 degrees and immediately photo-converted. The following day, immunostaining anti-myc (c, g) was performed on fish expressing Gal80 (a–d) or not expressing Gal80 (e–h). The figure depicts muscle fibers for the expression of Kaede^red^ (a, e) and Kaede^green^ (b, f). (Scale bars: 20 µm).

We tested whether Gal4 repression by Gal80 was dose-dependent. For this purpose, we generated a stable transgenic line *Tg[UAS:mem-TdTomato]* to express the red-fluorescent protein TdTomato in a Gal4-dependent manner. We co-injected eggs from this line with a cDNA coding for the full-length Gal4 under the transcriptional control of the beta-actin promoter for ubiquitous expression, together with different concentrations of Gal80 synthetic mRNA (Fig. 2A). At 3 dpf, embryos injected with 50 pg of Gal80 mRNA begun to express TdTomato in scattered muscle fibers (Fig. 2Ad). Animals from injections with 100 pg of Gal80 mRNA showed the same onset of TdTomato expression, but in fewer cells (Fig. 2Ag). Fish from injections with 200 pg of Gal80 mRNA, however, showed no fluorescence at 3 dpf (Fig. 2Aj). Starting at 4 dpf, all fish groups were strongly fluorescent but the 200 pg group continued to exhibit lower fluorescence (Fig. 2e–f, h–iA, k–l). In order to quantify the repressive activity of Gal80, we looked at the level of Td-Tomato expression at different time points in cells from fish injected with increasing concentrations of Gal80 RNA (Fig. 2B). For each condition, we selected individual cells and imaged them at 3, 4 and 6 dpf (Fig. 2Ba–i). We then quantified the fluorescence per µm^2^ using the ImageJ software (Fig. 2Bh). Comparisons between conditions confirmed our observations that the intensity of the red-fluorescence decreases with increasing amounts of Gal80 RNA. Following the same cells over time showed that red-fluorescence increased progressively, suggesting that Gal4-driven transgene expression increases following Gal80 degradation. The dose-dependent effect of Gal80 on the production of the fluorescent protein was confirmed in protein extracts from 3 dpf embryos and western blotting with an antibody to Td-Tomato (Fig. 3C). Without Gal80 RNA, we could detect a strong band corresponding to Td-Tomato (Figure 2Aa). Injections of 50 or 100 ng of Gal80 RNA weakened the signal, and we could not detect any Td-Tomato protein in extracts from fish injected with 200 ng of Gal80 RNA. Altogether, these observations suggest that Gal4 activity can be modulated or completely blocked by injecting increasing amounts of Gal80 RNA.

### Temporal control of Gal80 expression

Our previous approach offers to delay Gal4-driven gene expression during the first few days of development. However, we wanted to better control the timing of Gal80 activity. For this purpose, we created a transgenic line to express a Myc-tagged Gal80 under the control of a heat-shock promoter (Fig. 3A). The resulting *Tg[hsp70:Gal80-Myc]* stable line was then assessed by crossing it with *Tg[UAS:Kaede]* transgenics. The doubly-transgenic eggs were injected with beta-actin:Gal4 DNA. At 24 hpf, embryos expressing Kaede were subjected to a 30-minute or one-hour heat-shock at 39°C to activate Gal80 expression. To verify the expression of the Gal80 protein, we performed an anti-Myc western blotting (Fig. 3B). Without heat shock, we could not detect the Gal80 protein. After a 30-minute heat shock, we could detect a band at the correct size. The signal was stronger after 1-hour heat shock confirming that Gal80 expression is heat-shock dependent. We then looked at Gal80 expression in embryos by whole-mount *in situ* hybridization 8 hours after heat shock (Fig. 3Ca, d). In control embryos without heat-shock, we could not detect Gal80 RNA. By contrast, embryos submitted to the heat-shock displayed widespread expression of Gal80. To test if the Gal80-Myc was functional, we photo-converted the *Tg[hsp70:Gal80-Myc; UAS:Kaede]* embryos immediately after heat shock to distinguish the Kaede synthesized before the heat shock (Kaede^red^) from the Kaede synthesized after the heat shock (Kaede^green^). At 48 hpf,the embryos without heat shock were Kaede^red^ and stronglyKaede^green^ (Fig. 3Cb–c). By contrast after heat shock, the embryos were mainly red, indicating that Kaede expression was blocked by the heat-shock induced Gal80 (Fig. 3Ce–f). We also fixed 48 hpf embryos and stained them with an anti-Myc antibody to detect the Gal80 protein. Although the whole-mount *in situ* hybridizations showed ubiquitous expression of Gal80 mRNA, not every cell in the animal gave a Myc(+) signal, suggesting that expression of the Gal80-Myc fusion in the transgenic line is mosaic (data not shown). Cells expressing Gal80-Myc were mainly Kaede^red^ (Fig. 3D a–d). Low levels of Kaede^green^ may be due to incomplete photoconversion, or to Kaede protein translated from mRNA present at the time of photoconversion. By contrast, Gal80-Myc(-) cells were Kaede^red^ and intensely Kaede^green^ (Fig. 3De–h). Together, these results demonstrate that the stable *Tg[hsp70:Gal80-Myc]* transgenic line is able to inhibit Gal4 activity upon heat shock. Possitional effect silencing could explain the lack of ubiquitous expression in our *Tg[hsp70:Gal80-Myc]* line. This problem may be solved by generating additional lines carrying different insertion sites to select those expressing Gla80 in all cells. Another possibility to overcome positional effects over the transgene is to use insulator elements within the plasmid carrying the hsp70:Gal80-Myc.

### Delaying Gal4-VP16 activity with morpholinos

**Figure 4:**
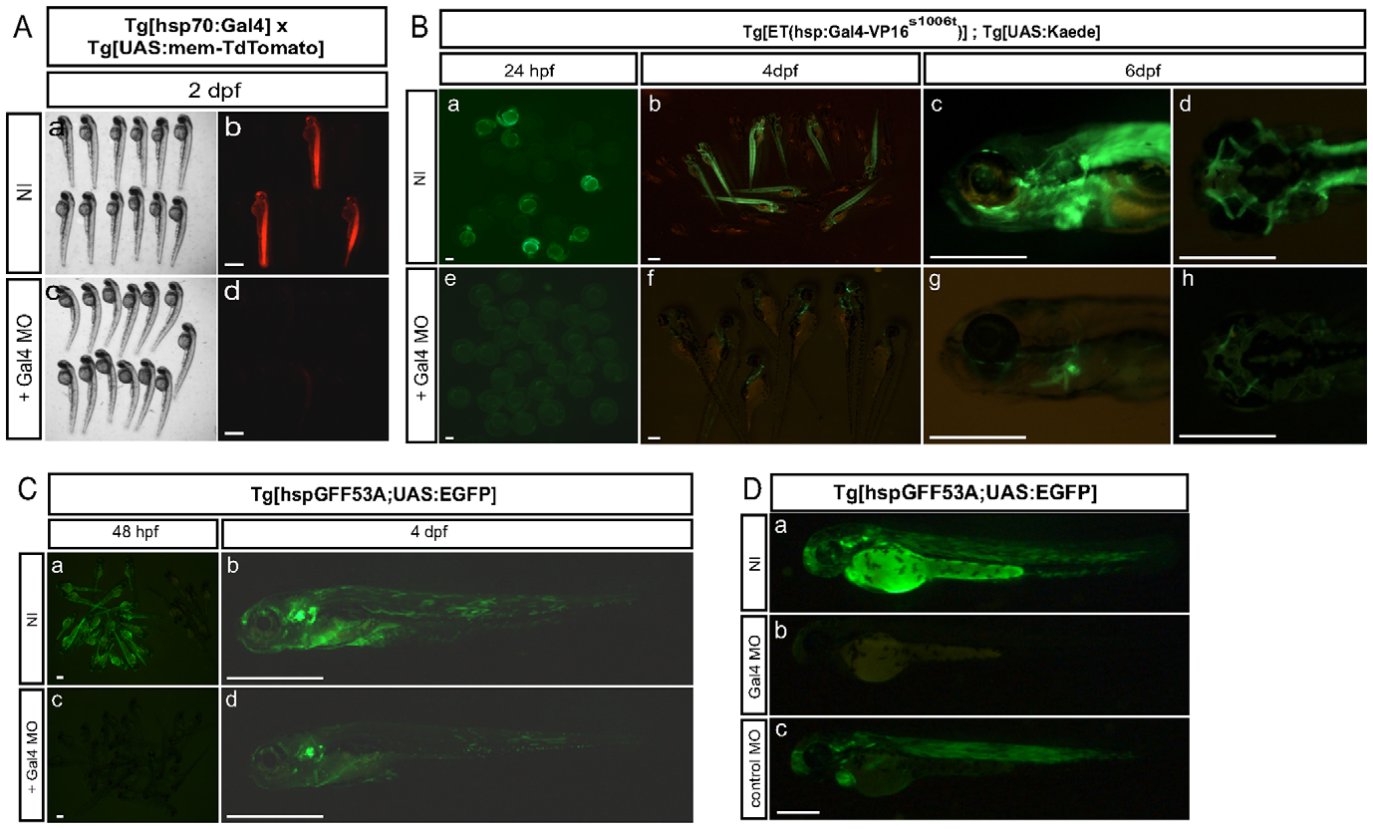
Gal4 morpholino temporarily inhibits Gal4 expression in Gal4-VP16 lines. (**A**) Embryos resulting from a cross between *Tg[hsp70:Gal4]* and *Tg[UAS:mem-TdTomato]* fish were either non-injected (NI, Aa-b, N = 27) or injected with 3 ng of Gal4 MO (Ac-d, N = 37). Representative specimens are depicted at 2 dpf. (**B**) Embryos resulting from a cross of *Tg[ET(hsp:Gal4VP16^s1006t^);UAS:Kaede]* double transgenic animals were either non-injected (NI, Ba-d, N = 47) or injected with 3 ng of Gal4 MO (Be-h, N = 52). Representative specimens are depicted at 24 hpf (Ba, e), 4 dpf (Bb, f) and 6 dpf (Bc-d, Bg-h). (**C**) Embryos resulting from a cross of *Tg[hspGFF53A;UAS:EGFP]* double transgenic animals were either non-injected (NI, Ca-b, N = 64) or injected with 3 ng of Gal4 MO (Cc-d, N = 58). Representative specimens are depicted at 48 hpf (Ca, c) and 4 dpf (Cb, d). (**D**). Embryos resulting from a cross of *Tg[hspGFF53A;UAS:EGFP]* double transgenic animals were either non-injected (NI, Da), injected with 5 ng of Gal4 MO (Db) or injected with 5 ng of a control MO (Dc). Representative specimens are depicted at 48 hpf. (Scale bars: 600 µm for A; 300 µm for B and C, 150 µm for D).

There are currently hundreds of stable transgenic lines expressing Gal4-VP16 in various cellular populations in nearly every organ. These lines bear an enormous potential for regenerative studies. However, the activation of Gal4-VP16 may not be controlled with the natural repressor because Gal80 antagonizes Gal4 by binding its C-terminal activation domain, which is missing in the Gal4-VP16 fusions [1]. As expected, we could not repress transgene activation with Gal80 in the two independent Gal4-VP16 lines that we tested (data not shown). Thus, we wanted to devise an alternative strategy to control the timing of Gal4-VP16 activity. For this purpose, we tested the ability of an antisense morpholino targeted to the first 25 coding nucleotides of the Gal4 gene to knock-down its translation. First, we injected 3 ng of a Gal4 morpholino (Gal4MO) into eggs from a cross between *Tg[hsp70:Gal4]* and *Tg[UAS:mem-TdTomato]* (Fig. 4A). At 24 hpf, the embryos were subjected to a 30-minute heat-shock at 39°C and looked for red fluorescence during the following day. The Gal4MO prevented the expression of mem-TdTomato (Fig. 4Ac–d), whereas the non-injected embryos displayed strong red fluorescence (Fig. 4Aa–b). Because the target sequence of the morpholino is shared between the native Gal4 and the Gal4-VP16 fusion, we tested the same morpholino in other transgenic lines (Fig. 4B–C). We first used the *Tg[ET(hsp:Gal4VP16^s1006t^)]* transgenics that express Gal4-VP16 at high levels in lateralis afferent ganglia, the ocular and trunk muscles and other tissues [13]. *Tg[ET(hsp:Gal4VP16^s1006t^);UAS:Kaede]* Gal4-morphants did not express Kaede for at least 72 hours after fertilization (Fig. 4Be). At 4 dpf, green fluorescence begun to appear (Fig. 4Bf). Kaede expression in these animals was restricted to ocular muscles, compared to a more widespread expression in non-injected specimens. This suggests that some early expression of the Gal4-VP16 is due to non-specific ubiquitous activation of the heat-shock promoter during embryogenesis. We also tested the Gal4MO in the *Tg[hspGFF53A]* transgenic line that carries the DNA-binding domain of Gal4 fused to two short transcriptional activation motifs of the VP16 designated Gal4FF [21] (Fig. 4C). The *Tg[hspGFF53A]* expresses the Gal4FF in afferent neurons of the ear and the lateral line, with background expression in axial muscle [22]. The injection of Gal4MO in eggs from the cross between the *Tg[hspGFF53A]* and *Tg[UAS:EGFP]* blocked green-fluorescence expression during the first 3 days of development (Fig. 4Cc). At 4 dpf, the fluorescence started to appear in the injected fish (Fig. 4Cd). The expression was restricted to the otic and lateralis afferent neurons with very low muscle expression. To confirm that this repressive effect was specific of the Gal4MO, we also injected these embryos with a control MO of which the first four base pairs are not complementary to the Gal4 sequence (Fig. 4D). Although the fluorescence was less intense than in the non-injected fish, we could detect strong expression of the EGFP in afferent neurons of the ear and the lateral line, and in axial muscle at 2 dpf, confirming the specificity of our Gal4MO.

**Figure 5:**
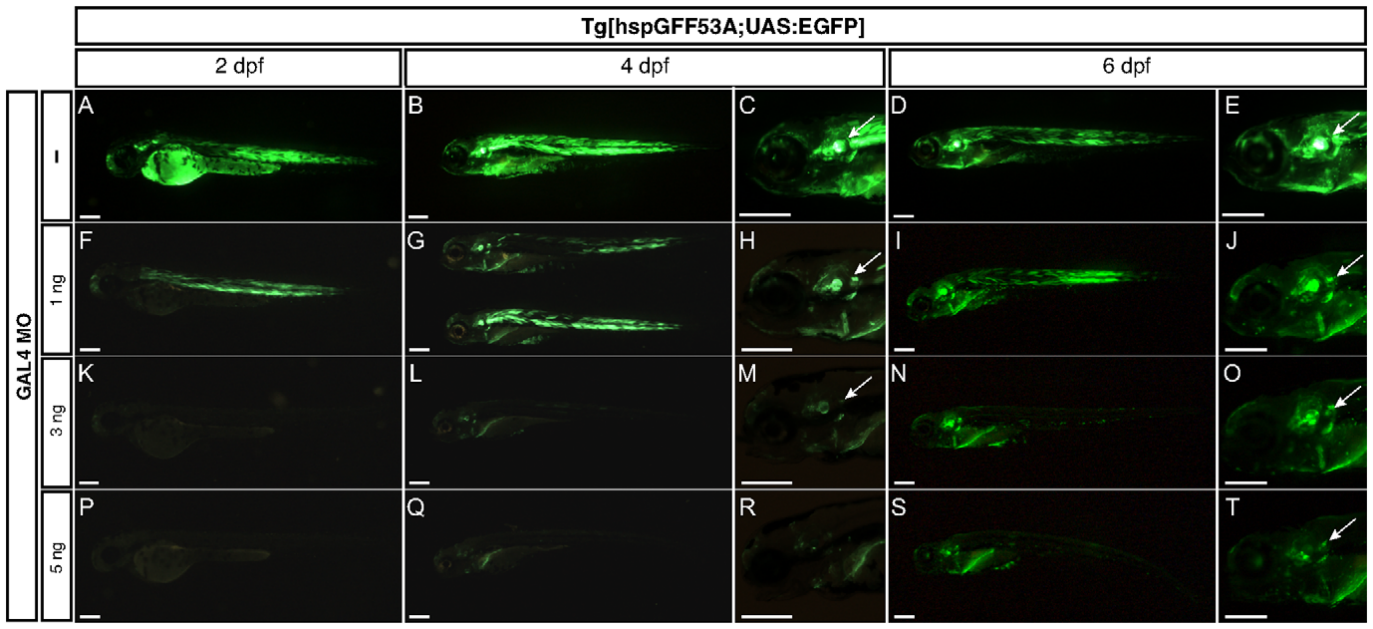
Gal4 morpholino temporarily inhibits Gal4 expression in a dose-dependant manner. Embryos resulting from a cross of *Tg[hspGFF53A;UAS:EGFP]* double transgenic animals were either non-injected (A–E) or injected with 1 ng (F–J), 3 ng (K–O) or 5 ng (P–T) of Gal4 MO (N = 94). Representative specimens are depicted at 2, 4 and 6 dpf. White arrows indicate green fluorescence at the level of the posterior afferent lateralis ganglion. (Scale bars: 150 µm).

We next tested whether Gal4 repression by the Gal4MO was concentration-dependent. For this purpose, we injected increasing amounts of Gal4MO in *Tg[hspGFF53A;UAS:EGFP]* double transgenic eggs and followed the green fluorescence over time (Fig. 5). Embryos injected with 1 ng of Gal4MO expressed EGFP at 2dpf (Fig. 5F). These animals showed fluorescence in the otic and posterior lateralis afferent ganglia at 4dpf (Fig. 5G–H), which increased in brightness by 6dpf (Fig. 5I–J). Injections of 3ng of Gal4MO produced fluorescent fish at 4 dpf, in which the EGFP signal was stronger and specific at 6 dpf (Fig. 5K–O). However, fish from eggs injected with 5 ng of the Gal4MO showed low EGFP even at 6 dpf (Fig. 5P–T). These data demonstrate that Gal4 and Gal4-VP16 activity can be repressed in a dose-dependant manner with morpholinos, and that the eventual release of this repression following morpholino degradation allows postembryonic expression of Gal4-driven transgenes.

### Delayed activation of Gal4-driven effectors bypasses tissue assembly

**Figure 6:**
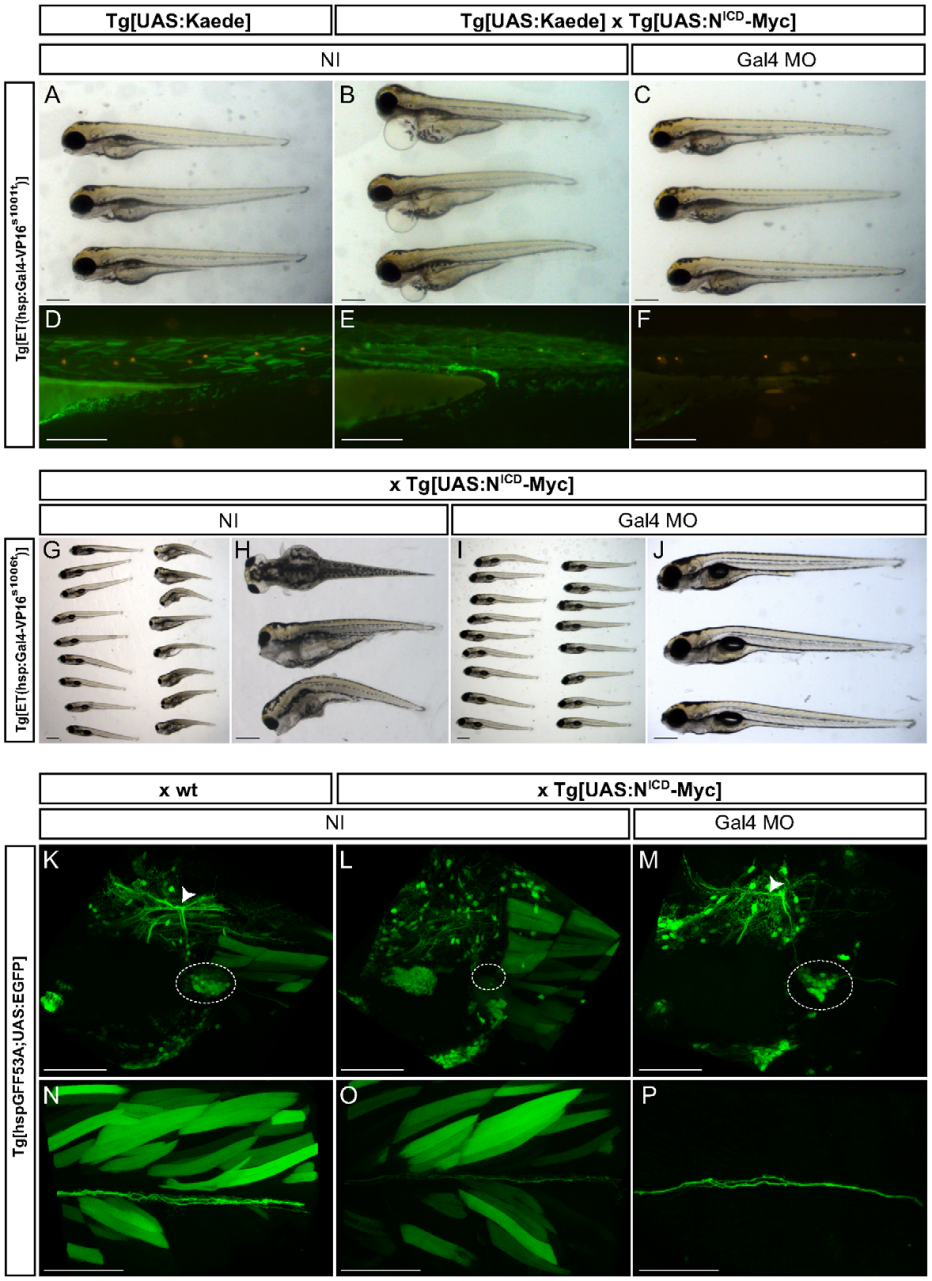
Gal4 morpholino allows bypassing of early developmental stages. (**A–F**) Embryos resulting from an incross between *Tg[ET(hsp:Gal4VP16^s1001t^);UAS:Kaede]* double trangenics (A,D) or from *Tg[ET(hsp:Gal4VP16^s1001t^);UAS:Kaede]* with *Tg[UAS:N^ICD^-Myc]* animals (B–C, E–F) were either non-injected (NI, A–B, D–E) or injected with 3 ng of Gal4 MO (C,F). Representative specimens are depicted at 3 dpf. The lower panel shows a magnification of the different specimens after DiASP treatment (D–F). (**G–J**) Embryos resulting from a cross between *Tg[ET(hsp:Gal4VP16^s1006t^)]* and *Tg[UAS:N^ICD^-Myc]* animals were either non-injected (G and magnification in H) or injected with 3 ng of Gal4 MO (I and magnification in J). Representative specimens are depicted at 6 dpf. (**K–P**) Embryos resulting from a cross between *Tg[hspGFF53A;UAS:EGFP]* double transgenic animals and either wt (K, N) or *Tg[UAS:N^ICD^-Myc]* (L–M, O–P) animals were either non-injected (K–L, N–O) or injected with 3 ng of Gal4 MO (M, P). Representative specimens are depicted at 7 dpf. (K–M) Maximal projections of posterior lateralis afferent ganglion (dashed circles) and central projection (white arrowheads). (N–P) Maximal projections of posterior lateralis afferent nerve. (Scale bars: 150 µm for A–F, H and J; 300 µm for G and I; 100 µm for K–P).

We wanted to further test the Gal4 morphant approach in animals expressing an effector transgene other than a fluorescent reporter. The motivation behind it is that UAS:X (where X is an effector gene that affects cellular fate acquisition or behavior) are powerful tools for *in vivo* functional analyses of organ homeostasis or regeneration. We chose to express a constitutively-activeform of the Notch receptor (N^ICD^) because Notch signalinghas been implicated in a multitude of biological processes [23], [24]. First, we crossed a *Tg[UAS:N^ICD^-Myc]* line to the *Tg[ET(hsp:Gal4VP16 s^1001t^);UAS:Kaede]* double transgenic line that expresses the Gal4-VP16 in the lateral-line neuromasts, the eyes, skeletal muscle fibers and several visceral organs [13] (Fig. 6A–F). *Tg[ET(hsp:Gal4VP16s^1001t^);UAS:Kaede;UAS:N^ICD^-Myc]* triple-transgenic embryos displayed multiple defects due to the unconditional activation of Notch signaling in many tissues during early development. At 3dpf, for example, they exhibited heart edemas and an inflated yolk sac (Fig. 6B). Animals carrying the hsp:Gal4VP16s^1001t^ or the UAS:N^ICD^-Myc transgenes alone showed none of these defects (Fig. 6A). Interestingly, when triple-transgenic larvae were immersed in DiASP to label lateral-line hair cells, we observed that they failed to incorporate the fluorophore in the neuromasts (Fig. 6D–E). When *Tg[ET(hsp:Gal4VP16s^1001t^);UAS:Kaede;UAS:N^ICD^-Myc]* eggs were injected with the Gal4MO, these defective phenotypes were absent (Fig. 6C,F), suggesting that the early inhibition of Gal4-VP16 translation with a morpholino is sufficient to bypass the critical developmental stages in which N^ICD^ induced these defects. We extended this approach to additional driver lines. We crossed a *Tg[UAS:N^ICD^-Myc]* line to the *Tg[ET(hsp:Gal4VP16s^1006t^)]* line (Fig. 6G–J). As we have shown, this driver line expresses the Gal4-VP16 in lateralis afferent ganglia, ocular and trunk nerves, but also has strong and variable background expression in muscle and the heart. At 6 dpf, doubly transgenic embryos showed a strongly reduced body length, smaller head and eyes, heart oedemas and inflated yolk sac (Fig. 6G–H). Again, when *Tg[ET(hsp:Gal4VP16s^1006t^); UAS:N^ICD^-Myc]* eggs were injected with the Gal4MO, these defective phenotypes were absent (Fig. 6I–J). We repeated the experiment crossing the *Tg[hspGFF53A;UAS:EGFP]* driver with the *Tg[UAS:N^ICD^-Myc]* effector (Fig. 6K–P). Triple transgenic animals displayed a very small posterior lateralis afferent ganglion compared to the *Tg[hspGFF53A;UAS:EGFP]* double-transgenics that served as control specimens (Fig. 6K–L). Afferent central projections were also reduced and there were fewer peripheral axons (Fig. 6N–O). However, when the triple transgenic eggs were injected with the Gal4MO, the central projections were normal, the posterior ganglion bore more cells and the number of peripheral axons increased (Fig. 6M,P). Furthermore, the expression of the EGFP and the N^ICD^-Myc transgenes were more specific. Indeed, we could not detect any background expression in axial muscle in Gal4MO injected fish (Fig. 6P). These results demonstrate that the Gal4 morphant approach is effective in suppressing the detrimental effect on tissue assembly of the early expression of effector transgenes. Interestingly, we found that in all stable Gal4-VP16 transgenic lines tested, the ensuing effector expression in the Gal4 morphants was more specific than that of control non-injected fish. One explanation for this difference is that these Gal4-VP16 transgenic lines use a minimal heat-shock promoter, which can induce non-specific early Gal4 expression. This non-specific expression may be reduced as animals age. Thus, the suppression of this initial background expression by the Gal4MO would result in the visualization of the later, specific expression of Gal4-VP16.

In conclusion, we have validated two strategies to delay Gal4 activity in the zebrafish. One is based on the Gal80 repressor. The second takes advantage of a morpholino to temporally knock-down the translation of Gal4 or Gal4-VP16. Both methods were effective in delaying Gal4-driven transgene activation. However, whereas the use of Gal80 is limited to transgenic lines expressing the native form of Gal4, the morphant approach can be easily applied to any Gal4-fusion transgenic line. Several large-scale enhancer-trap screens using various Gal4 constructs have recently been launched in the zebrafish [13], [14], [21], [25], [26], [27], [28], [29]. Therefore, the implementation of temporal control on these lines will add to their power. Because most of these lines carry an optimized version of the Gal4 that prevents the use of Gal80, the Gal4 morphant approach promises to become a standard strategy to delay transgene activation in studies of behavior, organ homeostasis or regeneration in the zebrafish.

## Materials and Methods

### Animals

Zebrafish were maintained under standardized conditions and experiments were conducted in accordance with protocols approved by the PRBB's Ethical Committee of Animal Experimentation. The reference from the Ethical Committee for Animals Research (CEEA) is HLS-08-111-I. Embryos were staged according to Kimmel et al. [30]. The *Tg[hspGFF53A;UAS:EGFP]* has been described previously [22]. The *Tg[ET(hsp:Gal4VP16s^1001t^)], Tg[ET(hsp:Gal4VP16s^1006t^)]* and *Tg[UAS:Kaede]* lines was obtained from Dr.H. Baier [29]. *The Tg[UAS:N^ICD^-Myc]* and *Tg[hsp70:Gal4]* have been previously described [24].

### Plasmid DNA constructs and injections

The hsp70:Gal80-myc, UAS:mem-TdTomato, β-actin:Gal4 and HuC:Gal4 constructs were obtained using the “Tol2 kit” [31]. Entry vectors were generated as described in the Invitrogen Multisite Gateway manual. PCR were performed using primers to add *att* sites onto the end of DNA fragments, using Platinum *Pfx* (Invitrogen).

For the generation of the middle entry clone containing the Gal80 cDNA (using pDONR 221), the forward PCR primer containing an *attB1* site and the reverse primer containing a reverse *attB2* site were used:

For: 5′-GGGGACAAGTTTGTACAAAAAAGCAGGCTCCACCATGGACTACAACAAGAGATC-3′

Rev: 5′-GGGGACCACTTTGTACAAGAAAGCTGGGTGTAAACTATAATGCGAGATAT-3′

For the generation of the middle entry clone containing the Gal4 cDNA (using pDONR 221), the forward PCR primer containing an *attB1* site and the reverse primer containing a reverse *attB2* site were used:

For: 5′- GGGGACAAGTTTGTACAAAAAAGCAGGCTCCACCATGAAGCTACTGTCTTCTAT-3′

Rev: 5′-GGGGACCACTTTGTACAAGAAAGCTGGGTGTTACTCTTTTTTTGGGTTTGGTG-3′

PCR products were purified using the Qiaquick gel extraction kit (Qiagen). BP and LR reaction were performed as described [31].

The pEntry vectors containing the hsp70 promoter, the myc tag, the UAS promoter and the β-actin promoter are from the “Tol2 kit” [31]. The pEntry vectors containing the TdTomato, the membrane targeting sequence and the HuC promoter have been described previously [32].

20 pg of the Tol2-expression clones and 20 pg of the transposase synthetic RNA were simultaneously injected into one-cell stage embryos.

To generate stable transgenic lines, injected embryos were raised to adulthood. For the *Tg[UAS:mem-TdTomato]* line, carriers were identified by crossing adult injected fish with *Tg[hsp70:Gal4].* Resulting embryos were submitted to a 30-minute heat shock and screened for Td-Tomato expression. Alternatively, the offspring was screened by genotyping using the following primers:

For: 5′-ACATGGCCGTCATCAAAGA-3′; Rev: 5′-CTTGTACAGCTCGTCCATGC-3′.

For the *Tg[hsp70:Gal80-myc]*, carriers were identified by genotyping their offspring using the following primers:

For: 5′-GTGGCCAGCCATTATGAAGT-3′; Rev: 5′-GGTAGGTTTGCCACCTTTGA-3′

**Morpholinos**

Morpholino oligonucleotides were obtained from Gene Tools (Philomath). The sequence of the morpholino to Gal4 is 5′-GTTCGATAGAAGACAGTAGCTTCAT-3′. The sequence of the control morpholino is 5′-ATAGAAGACAGTAGCTTCATGGTCC-3′. 1 to 5 ng of morpholino were injected into one-cell stage embryos.

### RNA synthesis and injections

5′ capped sense RNAs were synthesized using a construct encoding the transposase or the Gal80 cDNA [33] and the mMessage mMachine kit (Ambion). 20 pg and 50 to 200 pg of transposase RNA and Gal80 RNA respectively were injected in one to 2 cells-stage embryos.

### Labeling

For vital labeling of hair cells, zebrafish larvæ were immersed in a 500 µM solution of DiASP for three minutes at room temperature in the dark. Treated larvæ were washed briefly to remove excess fluorophore, anæsthetized in 3-aminobenzoic acid ethyl ester solution (Sigma), mounted on a glass slide, and aligned using a hair loop. For immunohistochemistry, larvae were fixed overnight at 4°C in a solution of 4% paraformaldehyde in phosphate-buffered saline (PBS) solution containing 1% Tween20. After fixation, samples were washed in the same solution without fixative and blocked at room temperature with 10% bovine serum albumin. Primary- and secondary-antibody incubations were conducted overnight at 4°C in PBS with 0.2% Tween 20. The primary mouse monoclonal anti-myc antibody was used at 1/50 (9E10.3, NeoMarkers). Alexa-Fluor 647 goat anti-mouse secondary antibody (Invitrogen) was used at 1/1000.

### Western blot

Embryos were anæsthetized in 3-aminobenzoic acid ethyl ester solution (Sigma), protein extracts from the whole embryos were prepared in 2X Laemmli sample buffer and loaded on SDS/PAGE. The primary rabbit polyclonal anti-RFP (Rockland) antibody was used at 1/1000. The primary mouse monoclonal anti-myc antibody was used at 1/1000 (9E10.3, NeoMarkers). Alexa-Fluor 680 goat anti-rabbit and anti-mouse secondary antibodies (Invitrogen) were used at 1/20000.

### Imaging

Kaede was photoconverted from green to red fluorescence by exposing the larvae to 405 nm light for 2 minutes. For low-resolution images, fish were mounted in 3% Methyl-cellulose and observed with a stereomicroscope (Leica MZ10F) and images were taken with a CCD camera (Leica DFC 490). For high-resolution imaging, fixed samples were mounted in Vectashield mounting medium (Vector Laboratories). Images were acquired with a Leica TCS SPE confocal microscope with a 40X oil immersion objective and Z-stacks were acquired at 0.8 or 0.5 µm intervals. Green (500–540 nm emission) and red (570–600 nm emission) fluorescence signals were captured by 488 and 532 nm laser lines. 3D reconstructions and cropping of Z-stacks were realized with Imaris software (Bitplane), pictures exported and processed in Adobe Photoshop and Illustrator softwares. The brightness and contrast of some pictures have been modified in order to help the reader eye but always in the same extend when it was calling for comparison.

### Fluorescence quantification

To quantify and compare the level of fluorescence, cells expressing TdTomato were imaged with the same parameters, avoiding saturation. Quantifications were performed using the Measure plug-in of the ImageJ software.

### Whole-mount *in situ* hybridization

We generated labelled RNA probes by *in vitro* transcription using the DIG/Fluor RNA labeling Mix (Roche). Embryos were fixed in 4% paraformaldehyde in PBS overnight at 4°C and whole-mount *in situ* hybridizations were carried as described by Thisse et al. [34]. The following antisense probe was used to characterize *gal80* expression: Saccharomyces cerevisiae S288c chromosome XIII (GenBank accession no. NC_001145), nucleotides 645–1308.
